# Impact of risk factors, activities and psychological disorders on the health of patients with chronic obstructive pulmonary disease in China: a cross-sectional study

**DOI:** 10.1186/1471-2458-13-627

**Published:** 2013-07-02

**Authors:** Lei Zhang, Peian Lou, Yanan Zhu, Peipei Chen, Pan Zhang, Jiaxi Yu, Ning Zhang, Na Chen, Hongmin Wu, Jing Zhao

**Affiliations:** 1 Xuzhou Center for Disease Control and Prevention, 142 West Erhuan Road, Xuzhou City 221006, Jiangsu Province, P. R. China; 2 Department of Respiratory Medicine, Affiliated Hospital of Xuzhou Medical College, 99 West Huaiai Road, Xuzhou City 221006, Jiangsu Province, P. R. China

**Keywords:** Chronic obstructive pulmonary disease, Health status, Survey

## Abstract

**Background:**

Patients with chronic obstructive pulmonary disease (COPD) often have organ dysfunction and resulting poor quality of life; however, in China little information is available regarding factors that affect their health. Here, the relationship between risk factors, activities and psychological disorders and health of patients with COPD in rural areas of Xuzhou, China was assessed.

**Methods:**

A cross-sectional study of 7597 COPD patients randomly selected by place of residence from 24,641 COPD patients who had been identified by screening of the 1.10 million health records of all residents of the target area was carried out to evaluate the relationships between risk factors, activities, psychological disorders and the ADO index (age, dyspnea, and airflow obstruction). The participants were assessed by spirometry and by administering a newly designed face-to-face questionnaire, which included items on general factors, risk factors, activities and psychological disorders. Correlations between the ADO index and the items addressed by the questionnaire were calculated.

**Results:**

The mean score of the ADO index was 3.7 ± 1.6. The ADO indices of current smokers, ex-smokers, and non-smokers were 3.9 ± 2.1, 3.7 ± 1.9, and 3.2 ± 1.5, respectively (*P* < 0.001). The ADO indices of cooks and non-cooks were 4.0 ± 2.2 and 3.5 ± 1.7, respectively (*P* < 0.001). The correlation coefficient between self-assessment of health status and ADO index was 0.976 (*P* < 0.001). Only 5.7% of patients reported no limitation of their daily living activities. The correlation coefficient between daily living activities and ADO index was 0.981 (*P* < 0.001). Only 5.5% of patients reported no limitation of social activities. The correlation coefficient between social activities and ADO index was 0.989 (*P* < 0.001), between the assessed anxiety score and ADO index 0.972 (*P* < 0.001), and between the assessed depression score and ADO index 0.989 (*P* < 0.001).

**Conclusions:**

COPD severity was significantly correlated with behavior (especially regarding smoking and cooking with biofuel in confined spaces), physical strength, daily living activities, social activities, anxiety and depression. Comprehensive approaches are required for the prevention and treatment of COPD.

## Background

Chronic obstructive pulmonary disease (COPD) is a progressive disease characterized by difficulties in airflow that are at least partially irreversible [1]. Smoking and air pollutants are both well-known risk factors for COPD [2]. COPD has a high incidence and mortality rate, being the seventh most common disease and the third most frequent cause of death worldwide [3,4]. The mortality rate of COPD ranges from 7.2 to 36.1 per 10^5^ individuals [5]. In China, over 40 million people have COPD, and the death toll is more than 1.28 million every year. Because of COPD, 5–10 million people are either partially disabled or completely unable to work and are thus unable to support themselves [6]. One study estimated that adults with COPD have a 10-fold greater risk of disability than those without COPD [7].

Recently, it has been recognized that COPD is a multisystem disease [8]. In addition to pulmonary-related symptoms such as limited airflow and dyspnea, COPD can indirectly affect a variety of organs, thereby resulting in functional disability [8], depression and anxiety [9], cognitive impairment [10], and decreased life quality [11]. In particular, activities of daily living can be severely impaired in patients with COPD owing to chronic psychological stress, somatic pain, and frequent hospital admissions.

COPD can cause a wide range of physical dysfunctions. Of the many symptoms of COPD, dyspnea, difficulties in daily living activities, physical mobility (exercise capacity), and anxiety and depression have the most direct impact on health status [12-14]. For instance, dyspnea often leads to reduced daily living activities, which in turn can exacerbate the dyspnea. Furthermore, dyspnea and fatigue are major factors that influence health-related quality of life. The interaction between progressive dyspnea and decreased health-related quality of life can lead to a worsened health status for patients with COPD [15].

Because clinical and patient-reported outcome measures such as dyspnea, exercise capacity, physical activity, exacerbation of symptoms, health status and mortality are an essential part of the clinical assessment of COPD [16], the objective of studies that assess the health of patients with COPD is to evaluate the relationships between these factors, reduced quality of life and COPD severity (assessed according to the ADO [(age, dyspnea, and airflow obstruction)] index) [2,17].

One limitation of only evaluating health status physiologically and clinically is that these measurements do not address patients’ social life, family and working relationships. These factors are important because they can influence both life quality and disease progression. Hence, health-related quality of life scores have also been used to evaluate the health status of COPD patients and the impact of their disease on them.

In the present study, we investigated risk factors, activities, psychological disorders and the general health status of COPD patients to elucidate correlations between daily living activities, risk factors, psychology and health status of patients with COPD. Our study clarifies how the severity of COPD impacts the daily lives of affected individuals.

## Methods

### Study design

A Basic Public Health Service survey mandating that all residents establish health records within 3 years was conducted in Jiangsu province, China from 2006. Tongshan county, in the Xuzhou city region of Jiangsu province, has 28 townships and 1.14 million inhabitants. From 1.10 million health records from this region screened by the end of 2007, 24,641 cases that fulfilled COPD diagnosis and treatment guidelines criteria were uncovered. Townships were assigned to investigation or not by simple randomization (a coin toss). Our study was conducted in association with the Basic Public Health Service. All selected patients were interviewed in their homes, analyzed for factors related to beliefs, treatments and economic burden that would make them vulnerable to further deterioration in health (18), and asked to undergo spirometry at health stations. This study was carried out over 3 months.

### Subjects

A flow chart describing the patient selection process has previously been published [18]. The following five entry criteria were used to select study participants: (i) age ≥ 40 years; (ii) maximal FEV_1_ (forced expiratory volume in one second)/forced vital capacity ratio < 0.7 and prebronchodilator FEV_1_ of < 80% of predicted value; (iii) registered at a local community health service center for > 6 months; (iv) no uncontrolled comorbidities; and (v) no COPD exacerbations over the preceding 6 weeks.

### Questionnaire design

A questionnaire aimed at assessing vulnerability to deterioration in health status was designed based on domestic and foreign literature [1,19-21]. It included 50 items divided into three domains: demographic (10 items), behavior (15 items) and quality of life (25 items). The quality of life domain included the following five categories.

The first quality of life category was self-assessment of physical strength, which was scored by the patients as follows: ‘0’, can be as active as an asymptomatic person without shortness of breath; ‘1’, shortness of breath with day-to-day work; ‘2’, shortness of breath when climbing stairs; ‘3’, shortness of breath with light activity; and ‘4’, shortness of breath when walking one hundred steps.

The second category, daily living activities, included 10 items, each scoring from 10 to 40 points as follows: 10 points, no limitation of daily living activities; 11–20 points, limitation of daily living activities; 21–30 points, severe limitation of daily living activities; and 31–40 points, extreme limitation of daily living activities.

The third item, social activities, included four items each scoring from 4 to 16 points as follows: 4 points, no effect on social activities; 5–8 points, some effect on social activities; 9–12 points, severe effects on social activities; and 13–16 points, extreme effects on social activities.

The fourth category, which dealt with the psychological symptoms of anxiety, included five items, each scoring from 5 to 20 points as follows: 5 points, no anxiety; 6–10 points, mild anxiety; 11–15 points moderate anxiety; and 16–20 points, severe anxiety. A score equal to or greater than 11 points was defined as anxiety.

The fifth category, psychological symptoms of depression, included five items, each scoring from 5 to 20 points as follows: 5 points, no depression; 6–10 points, mild depression; 11–15 points, moderate depression; and 16–20 points, severe depression. Patients with scores of 11 or more points were classified as depressed and those with scores of less than 11 points as not depressed.

For each of these 25 items there was a choice of four responses defining quantity or frequency of occurrence. The number of points assigned to each response corresponded to the number of the response. For example, response 1 scored one point, response 2 two points and so on. The total possible score was 100 points, higher scores indicating a worse quality of life as follows: 0–24 points, the patient felt very well; 25–49 points, the patient felt well; 50–75 points, the patient felt normal; and 76 points and above, the patient did not feel well. The questionnaire, which was designed by the present authors, has validity and reliability according to a pre-survey of COPD patients (unpublished data).

### Assessment of general characteristics

Home interviews were conducted with 8217 COPD patients. General characteristics based on information provided by the patients regarding age, sex, employment status, education, marital status, physical activity, smoking status, family COPD history, and other conditions were recorded.

The smoking status of each participant visited was classified as non-smoker, ex-smoker, or current smoker. Cigarette smoking was defined as having smoked at least 100 cigarettes during one's lifetime or having smoked continuously for at least 6 months [22]; if neither of these criteria were met, the participant was classified as a non-smoker. Those classified as current smokers included: (i) people who were smoking tobacco products at the time of the survey (including continual and intermittent smokers); and (ii) people who had quit but later restarted, or had quit for less than 6 months. Smoking cessation (former smoking) was defined as having not smoked tobacco products for at least 6 months. Passive smoking was defined as inhaling or being exposed to other people’s smoking at home or at work for at least 15 min per day for no fewer than 3 days per week.

Participants who were exposed to burning biomass through wood-fired stoves and cooked twice per day for at least 6 months were defined as cooks; otherwise, they were classified as non-cooks.

### Assessment of dyspnea

Dyspnea was assessed according to the Medical Research Council (MRC) dyspnea scale, which classifies breathlessness into six grades (0 to 5) according to self-perceived breathlessness during daily activities [23]. The MRC scale was translated by us and validated in our population (unpublished data).

### Pulmonary function

All patients were encouraged to undergo spirometry at health stations after completing their questionnaires. Spirometry and bronchodilator response tests were carried out by trained professionals according to the standardized guidelines of the American Thoracic Society [24]. Pulmonary function tests were performed at least 12 h after withdrawal of inhaled bronchodilators. Patients performed spirometry 15 and 60 min after inhaling salbutamol (200 μg) using a metered-dose inhaler with a spacer device.

### Assessment of COPD severity

The validated ADO index, which is a simple risk index, was used to evaluate COPD severity [17] and represent the pulmonary health status of patients with COPD. The ADO index is calculated based on age, score on MRC dyspnea scale, and FEV_l_ (forced expiratory volume in 1 s). The scores range from 0 (best status) to 5 (worst status) for age (with a greater point value the higher the age), 0–3 for MRC score, and 0–2 for FEV_l_. The points for each component are summed so that the ADO index ranges from 0 to 10 (higher scores indicating greater severity). The ADO index was also translated and validated in our population (unpublished data).

### Statistical analysis

Data analysis was performed using the Statistical Package for Social Science (SPSS) Version 13.0 software. Quantitative variables are expressed as means (along with the standard deviation [SD]) and qualitative variables as frequencies and percentages. The *χ*^2^ and Fisher exact tests were used to analyze the relationships between qualitative data. Analysis of variance was used for comparison of multiple samples. Questionnaire scores were correlated with the ADO index using correlation analysis. Statistical significance was set at a value of *P* < 0.05.

This study was approved by the Ethics Committee of the Xuzhou Center for Disease Control and Prevention and the Regional Ethical Vetting Board, Xuzhou, China. In addition, agreement was received from all of the relevant health centers. Informed consent was obtained from all participants in this study.

## Results

### General characteristics

**Figure 1:**
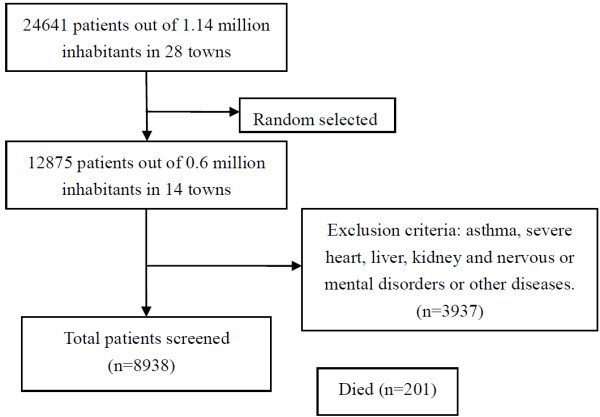
Sample random selection.

In this study, 8938 patients who met the criteria were selected to participate. Four hundred and thirty-five patients declined to take part in the survey or left town, and 201 patients died during the course of the study. The questionnaires of all the remaining 8302 patients were administered and returned; of these, 85 patients completed only part of the survey, leaving 8217 surveys that were complete and valid. Six hundred and twenty patients who were < 40 years old were excluded. The remaining 7597 acceptable questionnaires were analyzed; the response rate was 85.0% (see Figure 1). There were 3743 men and 3854 women. Patient ages ranged from 40 to 84 years (mean age, 62.21 ± 13.56 years). Disease severity determined according to the Global Initiative for Chronic Obstructive Lung Disease (GOLD) stage was distributed as follows: 4186 (55.1%) moderate, 2052 (27.0%) severe, and 1359 (17.9%) very severe.

### Chronic obstructive pulmonary disease severity

Table 1 lists the scores for the components of the ADO index. The median score for dyspnea was 1 (P5–P95, 0–3). The mean FEV_l_ was 1.25 L (SD, 0.35 L). The ADO index ranged from 0 to 9 with a mean score of 3.7 ± 1.6.

**Table 1:** **Scores for the components of the ADO indices of patients with COPD (*n*** **= 7597)**

**Variables value (label)**	**Number**	**Score**
FEV_1_(% predicted)		
0 (≥65%)	3213	0
1 (36 ~ 64%)	3339	3339
2 (≤35%)	1045	2090
Total	7597	5429
Dyspnea (MRC scale)		
0 (0)	698	0
0 (1)	2417	0
1 (2)	1904	1904
2 (3)	1479	2958
3 (4)	1099	3297
Total	7597	8159
Age (years)		
0 (40 ~ 49)	1224	0
1 (50 ~ 59)	1565	1565
2 (60 ~ 69)	2125	4250
3 (70 ~ 79)	2000	6000
4 (80 ~ 89)	683	2732
Total	7597	14547
ADO index	7597	28135

*FEV*_*1*_ Forced expiratory volume (1 s). *MRC* Medical research council.

### Smoking and cooking behavior of COPD patients

Of the 7597 patients with COPD, 6146 (80.9%) were smokers and of these 3692 were current smokers and the other 2454 patients ex-smokers. The ADO indices of current smokers, ex-smokers, and non-smokers were 3.9 ± 2.1, 3.7 ± 1.9, and 3.2 ± 1.5, respectively. There were significant differences among the three groups: current smoker vs. ex-smoker, t = 64.4, *P* < 0.001; current smoker vs. non-smoker, t = 192.0, *P* < 0.001; and ex-smoker vs. non-smoker, t = 8.6, *P* < 0.001.

Of the 7597 patients, 3426 (45.1%) cooked often or daily, and 1139 (33.2%) had been cooking for over 30 years. The ADO indices of cookers and non-cookers were 4.0 ± 2.2 and 3.5 ± 1.7, respectively. There was a significant difference between these two groups (t = 11.2, *P* < 0.001).

### The relationship between self-assessed physical strength and ADO index

Patients’ self-assessed physical strength is shown in Table 2. Only 799 (10.5%) patients showed a realistic understanding of their current health status. Further, there was a significant difference in the ADO index between every pair of groups (F = 575.0, *P* < 0.001). The correlation coefficient between self-assessment of health status and ADO index was 0.976 (t = 7.76, *P* < 0.001).

**Table 2:** Relationship between self-assessed physical strength and ADO index

**Variables**	**Total**	**Same as other asymptomatic people**	**Short of breath with common work**	**Short of breath when climbing stairs**	**Short of breath with light activities**	**Short of breath when walking for one hundred steps**
Self-assessed physical strength (n,%)	7597	799 (10.5%)	2465 (32.4%)	1855 (24.4%)	1429 (18.8%)	1049 (13.8%)
ADO index (mean ± SD)	3.7 ± 1.6	1.1 ± 1.1	1.6 ± 1.4	3.3 ± 1.6	5.9 ± 1.9	8.2 ± 2.7

### The relationship between activities of daily living and ADO index

**Figure 2:**
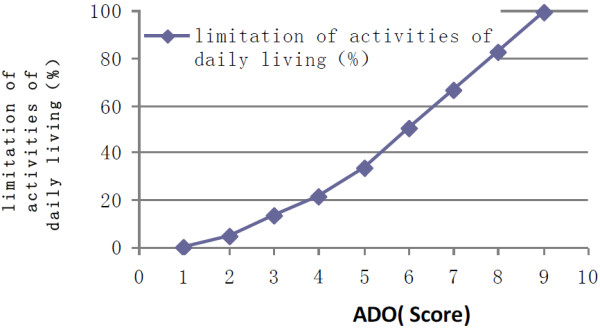
**Relationship between ADO score and limitation of activities of daily living (r = 0.981, t = 7.15, *P*** **< 0.001).**

The extent of limitations in daily living activities is shown in Table 3. Only 430 (5.7%) COPD patients reported no limitation in the 10 aspects of their daily living activities assessed by the survey. The correlation coefficient between daily living activities and ADO index was 0.981 (t = 7.15, *P* < 0.001) (Figure 2).

**Table 3:** Relationship between degree of limitation of activities of daily living and ADO index

**Activity**	**No limitation**	**Minor limitation**	**Major limitation**	**Severe limitation**
Dressing, washing, brushing teeth, combing, shaving, etc.	435 (5.7%)	1633 (21.5%)	3163 (41.6%)	2366 (31.1%)
Bathing	441 (5.8%)	1652 (21.7%)	3171 (41.7%)	2333 (30.7%)
Cooking, housekeeping, sweeping, laundry, etc.	461 (6.1%)	1728 (22.7%)	3201 (42.1%)	2207 (29.0%)
Outdoor walking	506 (6.7%)	1767 (23.3%)	3185 (41.9%)	2139 (28.1%)
Carrying goods	430 (5.7%)	1635 (21.5%)	3158 (41.6%)	2374 (31.2%)
Walking up stairs	431 (5.7%)	1681 (22.1%)	3173 (41.8%)	2312 (30.4%)
Appetite	456 (6.0%)	1712 (22.5%)	3198 (42.1%)	2231 (29.4%)
Sleep	432 (5.7%)	1641 (21.6%)	3186 (41.9%)	2338 (30.8%)
Taking medicine	516 (6.8%)	1731 (22.8%)	3231 (42.5%)	2119 (27.9%)
Entertainment (playing chess, playing cards, etc.)	439 (5.8%)	1637 (21.5%)	3153 (41.5%)	2368 (31.2%)
Assessment score (mean ± SD)	10 ± 0	13.7 ± 9.8	24.6 ± 13.2	35.4 ± 15.7
ADO index (mean ± SD)	1.4 ± 1.4	2.2 ± 1.6	3.3 ± 1.7	5.7 ± 2.1

### The relationship between social activities and ADO index

**Figure 3:**
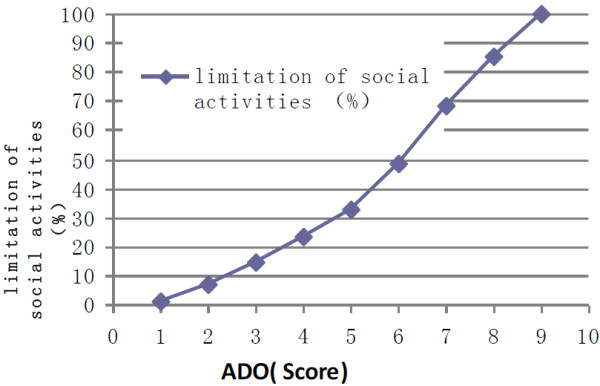
**Relationship between ADO score and limitation of social activities (r = 0.989, t = 9.31, *P*** **< 0.001).**

The extent of COPD patients’ limitations in social activities is shown in Table 4. Only 417 (5.5%) COPD patients reported no limitation in the four aspects of social activity assessed by the survey. The correlation coefficient between social activity and ADO index was 0.989 (t = 9.31, *P* < 0.001) (Figure 3).

**Table 4:** Relationship between degree of limitation of social activities and ADO index

**Activity**	**No limitation**	**Minor limitation**	**Major limitation**	**Severe limitation**
Communication with neighbors, colleagues, relatives and friends	428 (5.6%)	1713 (22.5%)	3019 (39.7%)	2437 (32.1%)
Making friends	435 (5.7%)	1682 (22.1%)	3115 (41.0%)	2365 (31.1%)
Joining neighborhood and public activities, etc.	417 (5.5%)	1637 (21.5%)	3145 (41.4%)	2398 (31.6%)
Relationship with family	478 (6.3%)	1745 (23.0%)	3267 (43.0%)	2107 (27.7%)
Assessment score (mean ± SD)	4 ± 0	6.8 ± 1.1	10.6 ± 2.3	14.7 ± 2.5
ADO index (mean ± SD)	1.2 ± 1.1	2.3 ± 1.5	3.5 ± 1.9	6.0 ± 2.7

### The relationship between anxiety and ADO index

**Figure 4:**
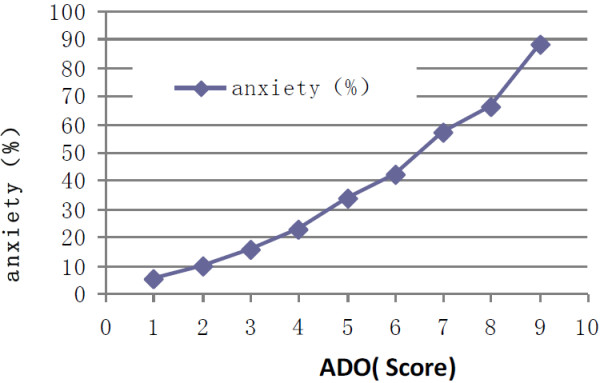
**Relationship between ADO score and anxiety (r = 0.972, t = 7.16, *P*** **< 0.001).**

According to our scoring system, 4335 patients scored 11 or higher, making their anxiety rate 57.1%. These results are shown in Table 5. The correlation coefficient between the assessed anxiety score and ADO index was 0.972 (t = 7.16, *P* < 0.001) (Figure 4).

**Table 5:** Relationship between degree of anxiety and ADO index

**Variables**	**No**	**Seldom**	**More**	**Often**^※^
Feeling easily fatigued	578 (7.6%)	2712 (35.7%)	3319 (43.7%)	988 (13.0%)
Feeling agitation	505 (6.6%)	2695 (35.5%)	3435 (45.2%)	962 (12.7%)
Feeling impatient (lost his/her temper for no reason)	593 (7.8%)	2521 (33.5%)	3299 (43.4%)	1184 (15.6%)
Feeling fear	712 (9.4%)	2627 (34.6%)	3351 (44.1%)	907 (11.9%)
Having nightmares	526 (6.9%)	2739 (36.1%)	3452 (45.4%)	880 (11.6%)
Assessment score (mean ± SD)	5 ± 0	7.3 ± 1.2	13.4 ± 2.5	17.4 ± 2.4
ADO index (mean ± SD)	1.1 ± 1.1	2.9 ± 1.4	4.2 ± 2.0	6.5 ± 2.8

※ Seldom, once per week; More, 2–3 times per week; Often, 4 or more times per week.

### The relationship between depression and ADO index

**Figure 5:**
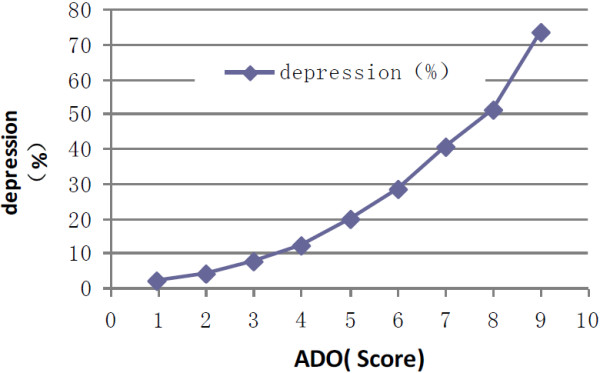
**Relationship between ADO score and depression (r = 0.989, t = 9.45, *P*** **< 0.001).**

According to our scoring system, 4335 patients scored 11 or higher, thus their rate of depression was 28.3%. These results are shown in Table 6. The correlation coefficient between the assessed depression score and ADO index was 0.989 (t = 9.45, *P* < 0.001) (Figure 5).

**Table 6:** Relationship between degree of depression and ADO index

**Variables**	**No**	**Seldom**	**More**	**Often**^※^	**total**
Depressed for sick	1276 (16.8%)	4023 (53.0%)	1522 (20.0%)	776 (10.2%)	7597
Feeling worthless	1377 (18.1%)	4104 (54.0%)	1360 (17.9%)	756 (10.0%)	7597
Feeling happy and satisfied with regards to life	1294 (17.0%)	4117 (54.2%)	1423 (18.7%)	763 (10.0%)	7597
Worrying about COPD affecting self-health	1192 (15.7%)	4165 (54.8%)	1453 (19.1%)	787 (10.4%)	7597
Feeling lonely	1416 (18.6%)	4009 (52.8%)	1392 (18.3%)	780 (10.3%)	7597
Assessment score (mean ± SD)	5 ± 0	7.5 ± 1.3	13.6 ± 2.4	17.6 ± 2.6	9.9 ± 2.0
ADO index (mean ± SD)	1.3 ± 1.2	3.2 ± 1.5	5.1 ± 2.1	7.2 ± 2.7	3.7 ± 1.6

※: Seldom, once per week; More, 2–3 times per week; Often, 4 or more times per week.

Classification of feelings of happiness or satisfaction: very satisfied; satisfied; fairly satisfied; dissatisfied.

## Discussion

This study is the first to analyze correlations between daily living activities, risk factors, psychological disorders and health status in Chinese COPD patients in the rural area of Xuzhou. We found that risk and psychological factors seriously impacted on COPD severity and reduced quality of life of these patients.

Several risk factors for COPD, namely smoking and cooking with biomass, were frequently present in the population assessed in this study [25]. Tobacco addiction is the best known cause of COPD [26]: 80% of COPD patients are current or ex-smokers [27,28]. A > 40-year-old person with a history of smoking > 20 packs per year has an increased risk of moderate to severe COPD [29]. Although quitting smoking is the most efficient way to prevent deterioration of lung function [30], 48.6% of patients with COPD in our study remained addicted to smoking. This rate is higher than that reported by Nuofu *et al*. [30] and lower than that reported by Eisner and his colleagues [31]. We also found that the ADO indices of the smokers were higher than that of the non-smokers, indicating that smokers had more severe COPD than did non-smokers [32].

Smoke generated by the burning of biomass is of particular relevance to COPD [33]. 45.1% of the patients in our study used biomass for cooking. This rate of biomass use is lower than that reported by Ran *et al*. [34]. We found that COPD patients who cook in this way more frequently are less healthy.

It is therefore critical to reduce the risk-related behaviors of patients with COPD. To this end, we should advocate cessation of smoking, reduction in frequency of passive smoking, the use of ventilation systems in kitchens, and reduction of the use of firewood.

The health-related life quality of patients with COPD deteriorates with increasing disease severity [35,36]. In addition, development of the non-respiratory impairments and dysfunctions that reflect the systemic nature of COPD appears to be a critical determinant of disability [12]. Moreover, patients with COPD have significantly reduced duration, intensity, and numbers of daily physical activity than do healthy control subjects [37]. Consistent with these findings, we found that COPD severity was significantly correlated with negative behaviors, physical strength, daily living activities, and social activities.

Our study also suggested a relationship between the vulnerability of patients to changes in mental health status (i.e. depression and anxiety) and their ADO index. Patients with COPD are at significantly higher risk of having depressive symptoms and their depression is associated with exacerbations of COPD and increased COPD-related hospitalization [38,39]. Moreover, anxiety is reportedly involved in diminished exercise performance, lower quality of life, and shortness of breath [13,40]. In the present study, we found that both depression and anxiety have significant correlations with COPD severity, which is consistent with previous studies.

This study is limited in that we primarily focused on farmers, who have such a poor awareness of COPD that they have difficulty in accurately reporting the symptoms of COPD. Thus, our results in this skewed population may have limited applicability in other parts of China or in other countries.

## Conclusions

In summary, our study demonstrated that COPD severity is significantly correlated with behavior, physical strength, daily living activities, social activities, anxiety and depression.

## Competing interests

The authors declare that they have no competing interests.

## Authors’ contributions

LZ and PL participated in writing the title and abstract, contributing to the writing of the manuscript drafts and reviewing the full text. YN conceptualized the study, participated in its design, title and abstract screening, full text screening, data extraction and analysis, and drafting of the manuscript. PC performed literature searches, participated in title and abstract screening, full text review, and contributed to the manuscript drafts. PZ and JY conceptualized the study; participated in its design, title and abstract screening, full text screening; and contributed to the manuscript drafts. NZ and NC contributed to the conception of the study, participated in the study design, and contributed to the manuscript drafts. HW and JZ were the lead authors of the original review, contributed to the conception of the study, participated in the study design, and contributed to the manuscript drafts. All authors have read and approved the final manuscript.

## Pre-publication history

The pre-publication history for this paper can be accessed here:

http://www.biomedcentral.com/1471-2458/13/627/prepub
